# Charting the content of data monitoring committee charters for clinical trials

**DOI:** 10.1177/17407745251389185

**Published:** 2025-12-10

**Authors:** Lisa Eckstein, Akram Ibrahim, Olivia Orr, Annette Rid, Seema K Shah

**Affiliations:** 1 Faculty of Law, University of Tasmania, Hobart, TAS, Australia; 2 Bellberry Limited, Adelaide, SA, Australia; 3 Ann & Robert H. Lurie Children’s Hospital of Chicago, Chicago, IL, USA; 4 Department of Bioethics, National Institutes of Health (NIH) Clinical Center, Bethesda, MD, USA; 5 Northwestern Feinberg School of Medicine, IL, USA

**Keywords:** Data monitoring committees, data monitoring, research ethics

## Abstract

**Background:**

Data monitoring committees play a critical role in ensuring the ethical conduct of clinical trials. Data monitoring committee charters set out the role and processes for data monitoring committees in monitoring clinical trials; however, little is known about the information charters contain.

**Methods:**

We conducted a summative content analysis of a convenience sample of data monitoring committee charters based on the criteria set out for charters by the DAMOCLES Study Group in 2005. Thirteen charters from public and commercially sponsored clinical trials were obtained for review.

**Results:**

Although the data monitoring committee charters we analyzed broadly satisfied the criteria set out by the DAMOCLES Study Group, some issues warrant further attention. These included variability in the availability of unmasked data for review, communication across data monitoring committees for related trials, post-trial DMC responsibilities, and a need for more explicit decision-making processes and conflict resolution procedures. Moreover, few of the data monitoring committee charters we were able to analyze included legal protection for members.

**Conclusion:**

Despite limitations due to the difficulties in obtaining data monitoring committee charters, the convenience sample reviewed suggests variability, including in terms of implementation of some best-practice recommendations. There is a need for further exploration of these issues in a larger sample size. Undertaking such research would be assisted by requiring or incentivizing public access to data monitoring committee charters.

## Introduction

Data monitoring committees (DMCs) play a critical role in ensuring the ethical conduct of clinical trials. Appointed by trial sponsors, DMCs gain exclusive access to interim trial data to make recommendations on whether the trial should stop, be modified, or continue. DMC charters set out the role and processes for clinical trial data monitoring.^1–3^

Although guidance about DMC charters is available,^4,5^ little is known about their implementation. To our knowledge, no available literature reports on the content of DMC charters. To fill this gap in the literature, we examined a convenience sample of DMC charters to review key components of their content based on the DAMOCLES Study Group^4^ proposed charter for clinical trial data monitoring committees. While the results are not drawn from a random sample and the sample size was small, they are a first step at filling this gap in the literature and suggest valuable directions for future research.

## Methods

We used an iterative approach to identifying charters. We initially sought a convenience sample from recently published Covid-19 and other infectious disease trials, which we expected to raise compelling monitoring challenges. Between January and May 2022, we contacted the corresponding author for 18 articles published in the influential medical journals *NEJM*, *JAMA* and *The Lancet* that reported results of Covid-19 trials. Of 18 trials identified, we received four charters.^†^

Because we only obtained a small number of charters from our initial strategy, we searched https://www.clinicaltrials.gov in August 2022. Trials were identified that were “interventional,” reported the availability of study results, and included a study protocol and statistical analysis plan. The results were manually searched for infectious disease trials that reported the use of a DMC. This identified a further nine trials. One principal investigator (PI) of these trials agreed to provide a charter.

Only one of the charters identified, however, was from a commercially sponsored clinical trial. To identify commercially sponsored clinical trial charters, we searched the legal database LexisNexis for charters obtained through the litigation document discovery process, given the role of lawsuits in unearthing commercial information.^6^ This afforded three additional charters, all of which related to commercially sponsored oncology trials. Five additional charters were identified incidentally to a separate, ongoing systematic literature review as supplementary materials to trial publications. Charters were named in accordance with whether they related to a public or commercially sponsored trial and their country of sponsorship (see Table 1). While this resulted in a collection of trial charters that varied based on the conditions and interventions being studied and the method by which they were sourced, it still allowed for an exploratory assessment of their key provisions.

**Table 1.** Key characteristics of available DMC charters.

DMC charter	Trial population	Experimental intervention(s)	Trial sponsor	Sponsor location
P-US1	Covid-19 outpatients	Convalescent plasma	Public	US
P-US2	Covid-19 outpatients	Antiplatelet agent	Public	US
P-US3	Ebola virus patients	Antiviral, monoclonal antibodies	Public	US
P-UK1	Covid-19 hospitalized patients	Antivirals, monoclonal antibodies, corticosteroids, convalescent plasma, anticoagulant, antibiotics (among others)	Public	UK
P-UK2	Malaria prevention	Long-lasting insecticidal nets	Public	UK
P-UK3	Dengue patients	Antiviral/immune modulator	Public	UK
P-EU1	Covid-19 hospitalized patients	Corticosteroid	Public	EU
P-AU1	Patients with severe injuries	Anti-fibrinolytic agent	Public	Australia
C-C1	Healthy individuals	Covid-19 vaccine	Commercial	China
C-US1	Breast cancer patients	Kinase inhibitor	Commercial	US
C-USC1	Renal cell cancer patients	Histone deacetylase (HDAC) inhibitor	Commercial	US/China
C-US2	Hepatocellular cancer patients	Anthracycline	Commercial	US
C-C2	Stroke patients	Serine protease	Commercial	China

The authors undertook a summative content analysis of the 13 available DMC charters based on categories drawn from the influential DAMOCLES Study Group^4^ proposed charter for clinical trial data monitoring committees. Table 2 sets out the categories of charter content we identified, alongside examples of ethically pertinent content. By this we mean content that pertains to respecting the rights and interests of trial participants and the intended beneficiaries of the research (e.g. future patients), as well as processes for making robust recommendations about such content. Authors L.E., A.I. and O.O. coded the charters using summative content analysis to examine the usage of specific words or content, including the range of meanings given to such words or content.^7^ Coding disagreements were resolved by discussion until consensus was reached.

**Table 2.** Key categories of DMC charter content and examples of ethically pertinent content.

Key categories of DMC charter content	Examples of ethically pertinent content
Roles and overall responsibilities	• Scope of DMC responsibilities (e.g. monitoring of safety, efficacy, recruitment)
Before the trial/early in the trial	• Review of the charter and trial protocol by DMC members • Indemnification of trial sponsor
Composition	• Expertise of DMC members • Consultation of additional experts
Relationships	• Protection against conflicts of interest of DMC members
Organization of meetings	• Meeting schedule (e.g. based on trial milestones)
Documentation and information sharing	• Data access by the DMC (e.g. masked vs unmasked data) • Protection of confidentiality (e.g. data, DMC deliberations) • Date or information sharing by the DMC (e.g. with similar ongoing trials)
Decision making	• DMC decision process (e.g. consensus, vote) • Process for managing disagreement among DMC members
Reporting	• Expectations for trial sponsors or leadership to respond to DMC recommendations (e.g. timeframe) • Process for managing disagreement between the DMC and trial sponsors or leadership • Confidentiality regarding DMC recommendations and whether they were followed
After the trial	• DMC input on trial results (e.g. in publications or regulatory submissions)

Source: DAMOCLES Study Group.^4^

## Results

Based on the iterative search strategy above, 13 DMC charters were available for analysis.

### Roles and overall responsibilities

Charters differed in the responsibilities assigned to the DMC. These included broad responsibilities to evaluate safety, efficacy, futility, and data quality (C-US1, C-USC1, P-US1, P-US2) and “to consider any issues or concerns raised by local investigators, funding bodies, etc that the Trial Steering Committee has not been able to resolve” (P-UK2). Several also included review of participant selection, recruitment, and retention (P-US1, P-US2, P-UK2, C-US1, C-USC1, P-US3). Although all thirteen charters permitted the DMC to review efficacy data, two specified a narrow scope of purview primarily focused on the review of safety data (P-US3C-US2).

With regard to statistical stopping guidelines, 2 of the 13 charters explained that guidelines were designed to reflect the anticipated strength of evidence needed to affect change in clinical practice. Ten included stopping boundaries that provided numerical information only and did not provide any additional guidance or principles for the DMC to follow. One commercial charter stated the DMC could recommend “termination of the trial at any point if it’s not ethical to continue,” leaving the DMC to determine what is “ethical.”

### DMC engagement before the trial/early in the trial

Five of the 13 charters expressly provided for a DMC member review of the charter either before joining the DMC or at the initial meeting (P-US2, C-C1, C-C2, P-UK2, P-UK3). Although the DAMOCLES Study Group recommended that DMC members should consider potential liability before the trial commences, only one charter (P-UK1) expressly mentioned sponsor indemnification of members.

### Composition

Eleven of the 13 available charters (with the exceptions of P-US1 and P-UK2) provided information about DMC member expertise. Of these, all required clinical and biostatistical expertise; one publicly sponsored trial also required “sufficient ethical expertise” (P-US2). Six of the charters allowed for the DMC to consult or appoint additional experts as needed, potentially including ethical expertise (P-UK1, P-US2, P-US3, C-USC1, C-US2, C-C1).

### Relationships

All 13 charters specified that the DMC was an advisory body to the trial sponsor and/or leadership group. One commercially sponsored charter specified that DMC members were to have no contact with trial investigators or participants; (C-US1) two publicly sponsored trials allowed for at least some contact with other parties, including reporting, if necessary, to the research ethics committee (P-UK2), and notifying the PI, in addition to the sponsor, of DMC findings “that require immediate action” (P-UK3).

With one exception (P-UK2), charters explicitly included protections against conflicts of interest among DMC members. Many included broad-ranging definitions, mostly focusing on financial conflicts (e.g. P-UK1, C-US1, C-C1, C-US2, P-EU1, P-AU1, C-C2). Other requirements for avoiding conflicts included maintaining “only necessary contact with the sponsor,” not having a “predetermined view” of the study, and avoiding authorship on academic papers related to study data (C-C2).

### Organization of meetings

Given the wide range of trials, DMC meeting schedules varied widely, from highly prescriptive timepoints for meetings through to more discretionary timetables (e.g. “normally once per year” (P-UK2)).

Ten of the 13 available charters specified a person responsible for providing statistical reports, 7 of which were allocated to an “independent” or “project” statistician. One publicly sponsored trial charter gave the PIs responsibility for preparing the statistical report. (P-UK2) Two charters for publicly sponsored trials were unclear about who would be responsible for preparing statistical reports. (P-EU1, P-UK1).

### Documentation and information sharing

All 13 charters reviewed explicitly protected the confidentiality of interim trial data and DMC deliberations, including through open and closed sessions. Only one charter expressly allowed for interim data to be shared with similar ongoing trials (P-UK1).

All 13 charters provided for DMCs to receive both efficacy and safety data at some or all meetings. One of the charters included review of efficacy as a DMC role but did not explicitly list efficacy information in the data to be produced for DMC meetings (C-C2).

Ten charters provided DMCs with access to unmasked (or “unblinded”) data. One publicly sponsored trial charter stated an “expectation” that the DMC would review unmasked data, but with DMC discretion to remain masked (P-US1). However, one commercially sponsored trial charter limited DMC review to masked data without providing for the DMC to request unmasked data (C-US1). One publicly sponsored trial charter specified a default of masked review, but the DMC could request unmasking from the Trial Management Committee (P-EU1).

### Decision making

The charters reviewed varied in the process for formulating DMC recommendations. Five emphasized that consensus was desirable, but voting could be used to make recommendations if consensus was not reached (P-UK1, P-UK3, C-US1, C-USC1, C-US2). Three adopted voting for decision making (P-US1, P-US2, C-C2). One included a process for managing disagreement by engaging an outside consultant or other actions acceptable to the parties involved (C-USC1). The remaining four charters did not include information on decision making.

### Reporting

Processes following a DMC recommendation were often unclear in the small sample of charters reviewed. Three required the trial sponsors or leadership to respond within a set timeframe (P-US3, P-EU1, C-USC1), two to inform trial sites (P-EU1, P-UK3), and four to inform responsible research ethics committees (C-C1, P-UK3, C-US2, C-C2). Two charters for commercially sponsored trials provided for regulators to be informed (C-US2, C-C2).

Only 2 of the 13 charters reviewed expressly dealt with disagreements between a DMC and the trial sponsors or leadership, both being charters for publicly sponsored trials. One provided for the appointment of an external arbitrator (P-UK2); another advised that “in the unlikely event of irreconcilable differences,” the DMC may decide to discontinue monitoring and disband (P-UK3). This charter further prohibited DMC members from making any public announcements about the sponsor’s final decision or the DMC’s recommendations (P-UK3).

### After the trial

DMC roles and responsibilities post-trial varied in the charters reviewed. Two charters provided for DMC input into the results manuscript (P-UK1) and into the drug regulatory authority submission (C-C1). In contrast, one advised that ‘At the conclusion of the trial the DSMB’s responsibilities for the studies cease’ (P-US3), presumably precluding such input. One stated that “All DMC members are expected to serve until the primary analysis is completed,” suggesting—with some ambiguity—this timepoint is when the DMC role terminates (P-US3). Finally, one charter advised that the final study report would be issued to the DMC, who may recommend continuing action items (P-UK3). The remaining eight charters were silent on post-trial roles.

## Discussion

In this small convenience sample, DMC charters of both publicly and commercially sponsored trials included content that broadly implemented the guidance by the DAMOCLES Study Group.^4^ Yet some issues warrant further attention in a larger study.

First, although most of the identified charters provided for the review of unmasked data, three suggested or required masked review. There is a strong, albeit not universal, position that DMCs should review unmasked data to promote timely insights about trial safety and the emerging risk-benefit profile of the study intervention.^8^ Viewing unmasked data can be particularly important if the trial results are unexpected.

Second, most charters restricted DMC communication to the sponsor or trial leadership. While the importance of maintaining confidentiality of interim data was reflected in charters, there is growing interest in the potential benefits from sharing interim results across related clinical trials.^9–11^ The only charter reviewed that expressly permitted this information-sharing was for a large adaptive Covid-19 trial, suggesting the need for further exploration of permissible information sharing in DMC charters, particularly those for large adaptive trials.

Third, in the small set of charters reviewed, we found variability in the recognition of DMC duties that should extend after the trial. The Covid-19 pandemic highlighted the challenges of accurately reporting trial results, and some high-profile conflicts occurred when DMC members publicly disagreed with the study team’s interpretation of interim findings.^12^ DMCs can play an important role in ensuring accuracy of dissemination about interim findings from trials that are stopped early, suggesting further attention on suitable DMC involvement post-trial.

Fourth, the charters we reviewed rarely included conflict resolution processes for when DMC members disagree with each other or with trial sponsors or leadership. Guidance on dealing with such disagreements is also limited,^5,13,14^ although the DAMOCLES charter requires decisions to be consensus-based where possible, with voting “only as a way of reaching consensus and after a full discussion.”^4^ On intra-DMC disagreement, many of the charters we reviewed relied on voting and 2 of the 13 provided no guidance whatsoever. Given that DMC disagreements can be a source of moral distress for members, attention to such processes is important.^15^ We found similarly sparse guidance for dealing with disputes between the DMC and the trial sponsor or leadership group. Further research is warranted on how such disputes are managed, including the use of mediators or other processes for resolving disagreements that could help with these situations.^16^

Finally, DMCs generally did not have legal protection in the charters we analyzed. The need for indemnification of DMC members has been raised as important,^17^ suggesting the need for further exploration in a larger sample of charters.

### Limitations

A paucity of DMC charters was yielded through requests to PIs; in particular, from commercially sponsored trials. Additional charters were sourced through publication or litigation to increase our sample size; however, this increased heterogeneity. Some information relevant to DMCs’ role and processes may have been in the trial protocol or statistical analysis plan.

## Conclusion

DMC charters play a crucial role in setting out the role and processes for clinical trial data monitoring. However, there is no literature reviewing these charters, potentially due to the difficulty of obtaining them. In our small sample of DMC charters, important issues were generally covered, but there was variability on some key issues. Some best-practice recommendations for charters are not widely implemented. Our review was limited by the difficulty of obtaining DMC charters. Requiring or incentivizing public access to DMC charters could help future research to better understand the practice of data monitoring.^2^

## Footnotes

† One additional charter was agreed to be provided only if we signed a confidentiality agreement.
